# Pythagorean  fuzzy deductive system of BCL-algebra

**DOI:** 10.12688/f1000research.159263.1

**Published:** 2025-01-10

**Authors:** Asmamaw Abebe Biabeyin, Berhanu Assaye Alaba, Yohannes Gedamu Wondifraw

**Affiliations:** 1 Mathematics, Bahir Dar University Department of Mathematics, Bahir Dar, Amhara, Ethiopia; 2 Mathematics, Bahir Dar University Department of Mathematics, Bahir Dar, Amhara, Ethiopia; 3 Mathematics, Bahir Dar University Department of Mathematics, Bahir Dar, Amhara, Ethiopia

**Keywords:** BCL-Algebra, Deductive system, Fuzzy set, Fuzzy Deductive system, Pythagorean Fuzzy Deductive system, Pythagorean Fuzzy set.

## Abstract

**Background:**

The deductive system of BCL-algebra has not been thoroughly explored, and its fuzzification remains undefined. This study aims to the association of this gap by introducing a fuzzy extension of the deductive system of BCL-algebra using Pythagorean fuzzy sets.

**Methods:**

We define a Pythagorean fuzzy set as a pair of membership and non-membership functions, where the sum of their squares lies between 0 and 1. These functions are used to extend the classical deductive system of BCL-algebra, allowing for reasoning based on fuzzy degrees. The system incorporates fuzzy operations such as union, intersection, and complement, while maintaining the structure of BCL-algebra. We also introduce key components such as the accuracy function, score function, degree of indeterminacy, and square deviation to measure the certainty, truth, uncertainty, and deviation of fuzzy sets.

**Results:**

We prove that the intersection of two Pythagorean fuzzy deductive systems remains a valid Pythagorean fuzzy deductive system within BCL-algebra. However, we show that the union of such systems does not necessarily form a valid fuzzy deductive system. The study also provides detailed proofs using induction, logical derivations, and algebraic techniques.

**Conclusion:**

The results disclose that while the intersection of Pythagorean fuzzy deductive systems preserves the system's structure, the union does not, offering new insights into the behavior and limitations of fuzzy systems in BCL-algebra.

## 1. Introduction

Georg Cantor (1874) ^3^ had introduced the concept of set theory (crisp set or a classical set) in mathematics as a fundamental theory and had defined a set as a collection of distinguishable and definite objects that contain elements which satisfy precise properties of membership. In such set theory, a subset U of a non-void set A could be defined by a characteristic function.

L. A. Zadeh (1965) ^20^ had introduced a generalization of classical sets called the concept of fuzzy set to handle mathematically vague or uncertain but Cantorian set could not address. A fuzzy set U in a non-empty set X is defined as: A = { ⟨x , $\mu_{U}\left(x\right)\rangle$ : x∈ A}, where the mapping $\mu_{U}$ : A → [0, 1] defines the degree of membership of the element x in A to set U. The values ‘0’ and ‘1’are used to represent complete non-membership and complete membership, respectively, and values in between ‘0’ and ‘1’ are used to represent intermediate degrees of membership.

As an extension of a fuzzy set, K. T. Atanassov (1986) ^1^ introduced the concept of an intuitionistic fuzzy set to better deal with uncertainties, later following K. T. Atanassov, R. R. Yager (2013) ^18^ launched Pythagorean fuzzy sets to alleviate the constraint on the total of membership degrees and non-membership degrees in intuitionistic fuzzy sets and introduced it as a new class of non-standard fuzzy subsets and the related idea of Pythagorean membership grades and non-membership grades.

An innovative algorithm stated by X. Fuyuan, D. Weiping ^10^ (2019) established by the Pythagorean fuzzy set distance degree is proposed to explain the problems of medical diagnosis. By involving the different methods in the medical diagnosis application, it is found that the new algorithm is capable of the other methods. These results ascertain that this method is practical in dealing with the medical diagnosis complications.

Y. H. Liu in 2011 ^14^ introduced notions of algebras, one of which is BCL-algebra, with the partial orders and different algebraic structures. However, in this algebra, deductive system of BCL-algebra has not been introduced nor has been fuzzifeid and hence we are initiated to fill these gaps, and further we are motivated to introduce the Pythagorean fuzzy deductive system of BCL-algebra in depth under this manuscript.

Thus in this paper, we define deductive system of BCL-algebra and then fuzzify it. We use the idea of Pythagorean fuzzy set to deductive system of in BCL-algebra. The concept of a Pythagorean fuzzy deductive system in BCL-algebra is given with some important characteristics. We have also examined the accuracy function, score function, degree of indeterminacy and square deviation in Pythagorean fuzzy deductive system of BCL-algebra and some interesting results have been delivered.

## 2. Preliminaries

Under this section, we recall some basic concepts of algebras, deductive systems and fuzzy deductive systems of a few algebras, discuss basic concepts of BCL-algebra and Pythagorean fuzzy sets that are related to our study.

Definition 2.1. (Ref. 11) A BCI-algebra is an algebra ( *X*; ∗ , 0) of type (2, 0) satisfying the following conditions; ∀x,y,z∈ *X.:*

(i)
x⊛x = 0,
(ii)
((x⊛y)⊛(x⊛z))⊛(z⊛y) = 0,
(iii)
(x⊛(x⊛y))⊛y = 0
(iv)
x⊛y = 0 and y⊛x=0⇒x=y .

Definition 2.2. (Ref. 5) An algebra ( *A,* ∗ , 0) is called a BCC-algebra if it satisfies the following axioms:

(1) ( ∀x *,* y *,* z∈ *A*)((( x∗y ) ∗ ( z∗y )) ∗ ( x∗z ) = 0),
(2) ( ∀x∈ *A*)(0 ∗x = 0),
(3) ( ∀x∈ *A*) ( x∗ 0 = x ),
(4) ( ∀x , y∈ A)(( x∗y = 0 and y∗x = 0) ⇒x = y ).

Definition 2.3. (Ref. 13) By a GE-algebra we mean a non-empty set *X* with a constant 1 and a binary operation “ ⊛ ” satisfying the following axioms:

(1)
u⊛u = 1,
(2) 1 ⊛u *=u,*
(3)
u⊛ ( v⊛w ) = u⊛ ( v⊛ ( u⊛w )), for all u , v , w∈ X.

Definition 2.4. (Refs. 4, 10) A Hilbert algebra *H* is an algebra ( *H*, ⊛ , 1) satisfying the following conditions; for all *x*, *y*, *z* in *H*:

(i)
x⊛ ( y⊛x ) = 1,
(ii) ( x⊛ ( y⊛z )) ⊛ (( x⊛y ) ⊛ ( x⊛z )) = 1,
(iii) if x⊛y = y⊛x = 1, then x = y .

Definition 2.5. (Ref. 2) A nonempty subset *D* of GE-algebra *X* is called a deductive system of *X* if it satisfies, ∀x *,* y *,* z∈ *X:*

(i) *X* ⊛ *D*:= { x⊛a *:, a* ∈ *D*} ⊆ *D.*
(ii) ( x , y∈ *D* ⇒ ( x⊛ ( y⊛z )) ⊛z∈ *D*).

Definition 2.6. (Ref. 9) A subset *A* of a Hilbert algebra *H* is called a deductive system of *H* if it satisfies; for all *x*, *y* in *H*:

(i) 1 ∈ *A*,
(ii)
x , x⊛y∈ *A* ⇒y∈ *A*.

Definition 2.7. (Refs. 15, 16) An algebra ( *B*; ⊛ , 0) of type (2, 0) is said to be a BCL-algebra if and only if for any x,y,z∈ *B*, the following conditions are satisfied:

(1)
x⊛x = 0,
(2)
x⊛y = 0 and y⊛x = 0 imply x=y ,
(3)
[((x⊛y)⊛z)⊛((x⊛z)⊛y)]⊛((z⊛y)⊛x) = 0.

Definition 2.8. (Ref. 17) Let ( *B*; ⊛ , 0) be a BCL-algebra. A binary relation ≤ on *B* by which x≤y if and only if x⊛y = 0 for any x,y∈ *B*, we call the BCL‐ordering ≤ is partial ordering on *B*.

Definition 2.9. (Ref. 6) Let x≤y if and only if x⊛y = 0. Then the definition for BCL-algebra above can be rewritten as:

(1)
x≤x ,
(2)
x≤y and y≤x⇒x=y ,
(3) [( x⊛y)⊛z]⊛ [( x⊛z)⊛y]≤ [( z⊛y)⊛x] .

Definition 2.10. (Ref. 21) A fuzzy set μ in a Hilbert algebra *H* is called a fuzzy deductive system of *H* if

(i)
μ (1) ≥μ ( *x*) for *x* ∈ *H,
*
(ii)
μ ( *y*) ≥ min {μ (x), μ ( *x* ⊛ *y*)} for all x , y∈ *H*.

Definition 2.11. (Ref. 7, 12) Let *X* ≠∅ . A fuzzy subset *A* of the set *X* is defined as: *A* = $\left\{\langle x,\mu_{A}\left(x\right)\rangle |x\in X\right\}$ , where the mapping $\mu_{A}\left(x\right)$ : *X* → [0, 1] defines the $\text{degree of membership and the complement of }\mu_{A}$ denoted by ${\bar{\mu }}_{A}$ , is the fuzzy set in *X* given by ${\bar{\mu }}_{A}\left(x\right)$ = 1 $-\mu_{A}\left(x\right)$ , for all x∈ *X*.

Definition 2.12. (Ref. 8, 11) An intuitionistic fuzzy set I in non-empty set *X* is an object having the form { x , $\mu_{I}\left(x\right)$ , $\nu_{I}\left(x\right)$ : x∈ *X*}, where the function $\mu_{I}\left(x\right)$ : *X* → [0, 1] and the function $\nu_{I}\left(x\right)$ : *X* → [0, 1] define the degree of membership and the degree of non−membership respectively satisfying the condition:

$$0\leq \mu_{I}\left(x\right)+\nu_{I}\left(x\right)\leq 1$$

Definition 2.13. (Ref. 19, 22) APythagorean fuzzysetP in a non-empty set *X* is an object having the form: P = {⟨x , $\mu_{P}\left(x\right)$ , $\nu_{P}\left(x\right)\rangle$ : x∈ *X* } or simply P = $(\mu_{P}$ , $\nu_{P})$ , where the function $\mu_{P}\left(x\right)$ : *X* → [0, 1] and $\nu_{P}\left(x\right)$ : *X* → [0, 1] define the degree of membership and the degree of non-membership, respectively satisfying the condition:

$$0\leq {\left(\mu_{P}\left(x\right)\right)}^{2}+{\left(\nu_{P}\left(x\right)\right)}^{2}\leq 1.$$

Definition 2.14. (Refs. 18, 22) Let P be Pythagorean fuzzy set. Then, the score functions of P is defined by:

$$s\left(P\right)={\left(\mu_{P}\left(x\right)\right)}^{2}-{\left(\nu_{P}\left(x\right)\right)}^{2},\;\text{where}\;s\left(P\right)\in [-1,1].$$

Definition 2.15. (Ref. 18) Let P be a Pythagorean fuzzy set. Then, the accuracy functiona of *P* is defined by:

$$a(P)={\left(\mu_{P}\left(x\right)\right)}^{2}+{\left(\nu_{P}\left(x\right)\right)}^{2}\;\text{and hence}\;a(P)\in [0,1]$$

## 3. Main Results

### 3.1 Pythagorean fuzzy BCL-structures on BCL-algebra

Under this section, we define deductive system of BCL-algebra which has not been defined so far under BCL-algebra and similarly we introduce fuzzy deductive system of the BCL-algebra and then discuss some properties and theorems on fuzzy deductive system of BCL-algebra accompanied by corresponding proofs. Furthermore, we introduce Pythagorean fuzzy deductive system in BCL-algebra, state and prove different properties and theorems which no one has tried, yet.

For this section; unless otherwise specified, *B*, *DS*. and $B^{P}$ denote “BCL-algebra ( *B*; ⊛ , 0)”, the word “deductive system”, and “Pythagorean fuzzy set ( $\eta_{P}$ , $\tau_{P}$ ) or $\left\{<x,\;\eta_{B}\left(x\right),\;\tau_{B}(x)>\;:x\in X\right\}$ ” respectively, where the functions $\eta_{P}\left(x\right)$ : *X* → [0, 1] and $\tau_{P}\left(x\right)$ : *X* → [0, 1] define the degree of membership and the degree of non-membership respectively , satisfying the condition: 0 $\leq {\left(\eta_{P}\left(x\right)\right)}^{2}$ + ${\left(\tau_{P}\left(x\right)\right)}^{2}\leq$ 1.

### 3.2 Deductive system − Some properties and fuzzy structures in BCL-algebra

Under this subsection, we define deductive systems of the BCL-algebra and fuzzy deductive systems of the BCL-algebra setting the stage for the subsequent development of Pythagorean fuzzy DS. of BCL-algebra where each concept is explained with examples for clarity.

Remark 3.1. For BCL-algebra, ( *B*; ⊛ *,* 0), one can easily prove that the following equations hold, ∀m , n∈ *B*:

(1) 0 ⊛m = 0,
(2)
m⊛n = m⇒m = 0,
(3)
m⊛n = n ⇒m = 0

Definition 3.2. A non-empty subset *D* of BCL-algebra ( *B*; ⊛ , 0) is called deductive system ( *DS*.) of *B* if it satisfies, ∀x *,* y *,* z∈ *B:*

(i)
x∈ *D* ⇒ ( x⊛z ) ⊛z∈ *D*,
(ii)
x , y∈ *D* ⇒x⊛ ( y⊛z ) ∈ *D*.

Example 3.3. Let *B* = {0, p , q , r } and define a binary operation ⊛ on *B* by the Cayley Table 1 as follows:

**Table 1.** A Cayley table of BCL-algebra, ( *B*; ⊛ , 0).

⊛	0	p	q	r
0	*0*	0	0	0
p	p	*0*	r	p
q	q	r	*0*	q
r	r	p	q	*0*

In the Table 1, it can be easily seen that *B* is a BCL-algebra and,

(1)
{0} , {0,r} , {0,p,q} and *B* are deductive systems of *B*
(2)
{p} , {q} , {r} , {0,p} , {0,q} , {p,q} , {p,r} , {q,r} , {0,q,r} , {0,p,r} , {p,q,r}

are not deductive systems of *B*.

Proposition 3.4. If *D* is deductive system of *B*, where ( *B*; ⊛ , 0) is BCL-algebra, then the following hold true:

(i) 0 ∈ *D*,
(ii) *D* has never exactly two elements.

Proof. Suppose *D* is deductive system of *B*.

(i) *D* ≠∅⇒∃x∈ *D* such that ( x⊛x ) ⊛x∈ *D* ⇒ 0 ⊛x = 0 ∈ *D.*
(ii) Let *D* = {0, x } ⇒x⊛ (0 ⊛z ) ∈ *D* ⇒x⊛ 0 ∈ *D*

⇒x⊛ 0 = 0 or x⊛ 0 = x

But by a remark above,

x⊛ 0 = 0 ⇒x = 0 and also, x⊛ 0 = x⇒x = 0

⇒ in any case, *D* = {0} ◻

Definition 3.5. A fuzzy set $\eta_{B}$ in a BCL-algebra *B* is called a fuzzy deductive system (fuzzy *DS*.) of *B*, if the following axioms are satisfied, for all x , y , z∈ *B*:

(i)
$\eta_{B}\left(\left(x⊛y\right)⊛y\right)\geq \eta_{B}\left(x\right)$

(ii)
$\eta_{B}\left(x⊛\left(y⊛z\right)\right)\geq \min\{\eta_{B}\left(x\right)$ , $\eta_{B}\left(y\right)\}$

Example 3.6. Suppose *B* = {0, q , r , p } and the binary operation ⊛ on *B* is as given by the Table 1 above:

Define a fuzzy set $\eta_{B}$ : *B* → [0. 1] by: $\eta_{B}\left(x\right)$ = $\left\{ \begin{array}{l} 0.8,\;\text{if}\;x=0,\\ 0.6,\;\text{if}\;x=p,q,\\ 0.2,\;\text{if}\;x=r. \end{array}\right.$

Then it is easy to check that $\eta_{B}$ is a fuzzy *DS*. of the BCL-algebra, *B*.

### 3.3 Pythagorean fuzzy deductive system of BCL-algebra

Definition 3.7. A Pythagorean fuzzy set $B^{P}$ = $(\eta_{P}$ , $\tau_{P})$ , where the functions:

$\eta_{P}\left(x\right)$ : *B* → [0, 1] and $\tau_{P}\left(x\right)$ : *B* → [0, 1] define the degree of membership and the degree of non‐membership , respectively in B is called a Pythagorean fuzzy *DS*. of *B* if the following axioms are satisfied, for all x , y , z∈ *B*:

(i)
${\left(\eta_{B}\left(\left(x⊛y\right)⊛y\right)\right)}^{2}\geq {\left(\eta_{B}\left(x\right)\right)}^{2}\;$ and $\;{\left(\tau_{B}\left(\left(x⊛y\right)⊛y\right)\right)}^{2}\leq {\left(\tau_{B}\left(x\right)\right)}^{2}$

(ii)
${\left(\eta_{B}\left(x⊛\left(y⊛z\right)\right)\right)}^{2}\geq \min\{{\left(\eta_{B}\left(x\right)\right)}^{2}$ , ${\left(\eta_{B}\left(y\right)\right)}^{2}\}\;$ and $\;{\left(\tau_{B}\left(x⊛\left(y⊛z\right)\right)\right)}^{2}\leq \max\{{\left(\tau_{B}\left(x\right)\right)}^{2}$ *,* ${\left(\tau_{B}\left(y\right)\right)}^{2}\}$

Example 3.8. Let *B* and the binary operation ⊛ be as defined as in Table 1 and let the fuzzy sets $\eta_{B}$ : *B* → [0. 1] and $\tau_{B}$ : *B* → [0. 1] be defined as follows:

$$\eta_{B}\left(x\right)=\left\{ \begin{array}{l} 0.83,\;\text{if}\;x=0,\\ 0.54,\;\text{if}\;x=p,q,\\ 0.16,\;\text{if}\;x=r \end{array}\right.\;\text{and}\;\tau_{B}\left(x\right)=\left\{ \begin{array}{l} 0.24,\;\text{if}\;x=0,\\ 0.67,\;\text{if}\;x=p,q\\ 0.76,\;\text{if}\;x=r \end{array}\right.$$

Then by routine calculations, it can be easily seen that $B^{P}$ = $\left(\eta_{B},\tau_{B}\right)$ is a Pythagorean fuzzyDS. of *B* (butnot intuitionistic fuzzyDS.ofB) .

Lemma 3.9. Let $B^{P}$ = $(\eta_{B}$ , $\tau_{B})$ be Pythagorean fuzzy set in *B*. If $B^{P}$ is Pythagorean fuzzy *DS*. of *B*, then the following hold, ∀m , n , u∈ *B*:

(i)
${\left(\eta_{B}\left(0\right)\right)}^{2}\geq {\left(\eta_{B}\left(m\right)\right)}^{2}\;$ and $\;{\left(\tau_{B}\left(0\right)\right)}^{2}\leq {\left(\tau_{B}\left(m\right)\right)}^{2}$

(ii)
m⊛n *=* $n\Rightarrow {\left(\eta_{B}\left(u\right)\right)}^{2}\geq {\left(\eta_{B}\left(m\right)\right)}^{2}\;$ and $\;{\left(\tau_{B}\left(u\right)\right)}^{2}\leq {\left(\tau_{B}\left(m\right)\right)}^{2}$ (Independent of n )
*And,* ${\left(\eta_{B}\left(u\right)\right)}^{2}\geq$ min{ ${\left(\eta_{B}\left(m\right)\right)}^{2}$ *,* ${\left(\eta_{B}\left(n\right)\right)}^{2}$ } and $\;{\left(\tau_{B}\left(u\right)\right)}^{2}\leq$ max{ ${\left(\tau_{B}\left(m\right)\right)}^{2}$ *,* ${\left(\tau_{B}\left(n\right)\right)}^{2}$ }
(iii)
m⊛n *=* $n⊛m\Rightarrow (\eta_{B}($ ( *m* ⊛ *u*) ${⊛u))}^{2}\geq {\left(\eta_{B}\left(u\right)\right)}^{2}$ and $\;(\tau_{B}($ ( *m* ⊛ *u*) ${⊛u))}^{2}\leq {\left(\tau_{B}\left(u\right)\right)}^{2}$

Proof. Let $B^{P}$ = $(\eta_{B}$ *,* $\tau_{B})$ be Pythagorean fuzzy *DS.* in *B*.

(i) Straight forward.
(ii) Let m⊛n *=* n

$\Rightarrow {\left(\eta_{B}\left(m⊛u\right)⊛u\right)}^{2}\geq {\left(\eta_{B}\left(m\right)\right)}^{2}\;$ and ${\left(\tau_{B}\left(m⊛u\right)⊛u)\right)}^{2}\leq {\left(\tau_{B}\left(m\right)\right)}^{2}$

$\Rightarrow {\left(\eta_{B}(u)\right)}^{2}\geq {\left(\eta_{B}\left(m\right)\right)}^{2}\;$ and $\;{\left(\tau_{B}\left(u\right)\right)}^{2}\leq {\left(\tau_{B}\left(m\right)\right)}^{2}$

(iii) Let m⊛n *=* n⊛m

(Independent of n since: ${\left(\eta_{B}\left(m⊛u)⊛u\right)\right)}^{2}$ *=* ${\left(\eta_{B}\left(u⊛\left(m⊛u\right)\right)\right)}^{2}$ *=* ${\left(\eta_{B}\left(u⊛\left(u⊛m\right)\right)\right)}^{2}$

≥ min $\left\{{\left(\eta_{B}\left(u\right)\right)}^{2},{\left(\eta_{B}\left(u\right)\right)}^{2}\right\}\;$ *=* ${\left(\eta_{B}\left(u\right)\right)}^{2}$ , and

$\Rightarrow {\left(\tau_{B}\left(m⊛u)⊛u\right)\right)}^{2}\leq {\left(\eta_{B}\left(u\right)\right)}^{2}$ (Independent of n ) ◻

Definition 3.10. For membership fuzzy set; $\mu_{B}$ *: B* → [0, 1], and square subtrahend

${\bar{\bar{\mu }}}_{B}$ : *B* → [0, 1] from 1 such that ${\left({\bar{\bar{\mu }}}_{B}\left(m\right)\right)}^{2}$ = 1 $-{\left(\mu \left(m\right)\right)}^{2}$ , we call such fuzzy set, ${\bar{\bar{\mu }}}_{B}$ ,

the square deviation of $\mu_{B}$ .

Theorem 3.11. Let $\eta_{B}$ and its square deviation ${\bar{\bar{\eta }}}_{B}$ be fuzzy sets in *B* such that

$\;{\left(\eta_{B}\left(m⊛n\right)\right)}^{2}$ = ${\left(\eta_{B}\left(n\right)\right)}^{2}$ . Then $\;{\left({\bar{\bar{\eta }}}_{B}\left(m⊛n\right)\right)}^{2}$ = ${\left({\bar{\bar{\eta }}}_{B}\left(n\right)\right)}^{2}$ , ∀m , n∈ *B*.

Then, $B^{P}$ = $(\eta_{B}$ , ${\bar{\bar{\eta }}}_{B})$ is Pythagorean fuzzy *DS*. of *B*. iff $\eta_{B}$ and then $\;{\bar{\bar{\eta }}}_{B}$ are constants.

Furthermore, $\mathrm{he}\;\text{accuracy function}\;{\text{a}}_{B}\left(m\right)$ , the $\text{score function}\;s_{B}\left(m\right)$ and the degree of

$\text{indeterminacy}\;\pi_{B}\left(m\right)$ are respectively given as: ∀m∈ *B*:

(a)
$a_{B}\left(m\right)$ = 1,
(b)
$s_{B}\left(m\right)$ = 2 ${\left(\eta_{B}\left(m\right)\right)}^{2}-$ 1,
(c)
$\pi_{B}\left(m\right)$ = 0.

Proof. Suppose ${\left(\eta_{B}\left(m⊛n\right)\right)}^{2}$ = ${\left(\eta_{B}\left(n\right)\right)}^{2}$

Then ${\left({\bar{\bar{\eta }}}_{B}\left(m⊛n\right)\right)}^{2}$ = 1 $-{\left(\eta_{B}\left(m⊛n\right)\right)}^{2}$ = 1 $-{\left(\eta_{B}\left(n\right)\right)}^{2}$ = ${\left({\bar{\bar{\eta }}}_{B}\left(n\right)\right)}^{2}\;$ and

Let $B^{P}$ = $(\eta_{B}$ , ${\bar{\bar{\eta }}}_{B})$ be Pythagorean fuzzy *DS*.. of *B*, a nd then ${\left({\bar{\bar{\eta }}}_{B}\left(m⊛n\right)\right)}^{2}$ = ${\left({\bar{\bar{\eta }}}_{B}\left(n\right)\right)}^{2}$ ,

Now we claim to verify that $\eta_{B}$ and ${\bar{\bar{\eta }}}_{B}$ are constants,

( or ${\left(\eta_{B}\left(m\right)\right)}^{2}$ = ${\left(\eta_{B}\left(n\right)\right)}^{2}$ and then ${\left({\bar{\bar{\eta }}}_{B}\left(m\right)\right)}^{2}$ = ${\left({\bar{\bar{\eta }}}_{B}\left(n\right)\right)}^{2}$ , ∀m , n∈ *B*. )

As $B^{P}$ = $(\eta_{B}$ , ${\bar{\bar{\eta }}}_{B})$ is a Pythagorean fuzzy *DS*. of *B*, $\eta_{B}$ is a fuzzy *DS*. of *B*, and hence by

one of the axioms, we have $\eta_{B}\left(0\right)$ = $\eta_{B}\left(0⊛m\right)$ , ∀m∈ *B*,

$\Rightarrow {\left(\eta_{B}\left(0\right)\right)}^{2}$ = ${\left(\eta_{B}\left(\left(0⊛m\right)⊛m\right)\right)}^{2}$ = ${\left(\eta_{B}\left(m\right)\right)}^{2}$ , ∀m∈ *B*, and again

$\;{\left(\eta_{B}\left(0\right)\right)}^{2}$ = ${\left(\eta_{B}\left(\left(0⊛n\right)⊛n\right)\right)}^{2}$ = ${\left(\eta_{B}\left(n\right)\right)}^{2}$ , ∀n∈ *B*

$\Rightarrow {\left(\eta_{B}\left(0\right)\right)}^{2}$ = ${\left(\eta_{B}\left(m\right)\right)}^{2}$ = ${\left(\eta_{B}\left(n\right)\right)}^{2}$ , ∀m , n∈ *B*,

Or ${\left(\eta_{B}\left(m\right)\right)}^{2}$ = ${\left(\eta_{B}\left(n\right)\right)}^{2}$ , ∀m , n∈ *B* and hence, $\eta_{B}$ is constant, and analogously, ${\bar{\bar{\eta }}}_{B}$ is, too.

Conversely, suppose $\eta_{B}$ and ${\bar{\bar{\eta }}}_{B}$ are constants, or: $\eta_{B}\left(m\right)$ = $\eta_{B}\left(n\right)$ and ${\bar{\bar{\eta }}}_{B}\left(m\right)$ = ${\bar{\bar{\eta }}}_{B}\left(n\right)$ , ∀m , n∈ *B*

${\left(\eta_{B}\left(m⊛n\right)\right)}^{2}$ = ${\left(\eta_{B}\left(n\right)\right)}^{2}$ and ${\left({\bar{\bar{\eta }}}_{B}\left(m⊛n\right)\right)}^{2}$ = ${\left({\bar{\bar{\eta }}}_{B}\left(n\right)\right)}^{2}$ , and

To prove: $B^{P}$ = $(\eta_{B}$ , ${\bar{\bar{\eta }}}_{B})$ is a Pythagorean fuzzy *DS*. of *B*,

(i)
${\left(\eta_{B}\left(m\right)\right)}^{2}$ = ${\left(\eta_{B}\left(n\right)\right)}^{2}$ = ${\left(\eta_{B}\left(\left(m⊛n\right)⊛n\right)\right)}^{2}$

$\Rightarrow {\left(\eta_{B}\left(\left(m⊛n\right)⊛n\right)\right)}^{2}\geq {\left(\eta_{B}\left(m\right)\right)}^{2}$ ,

( and then $\left\{{\left({\bar{\bar{\eta }}}_{B}\left(\left(m⊛n\right)⊛n\right)\right)}^{2}\geq {\left({\bar{\bar{\eta }}}_{B}\left(m\right)\right)}^{2}\right)$

(ii)
${\left(\eta_{B}\left(m⊛\left(n⊛u\right)\right)\right)}^{2}$ = ${\left(\eta_{B}\left(m\right)\right)}^{2}$ = ${\left(\eta_{B}\left(n\right)\right)}^{2}$ = ${\left(\eta_{B}\left(u\right)\right)}^{2}\geq$ min $\{{\left(\eta_{B}\left(m\right)\right)}^{2}$ , ${\left(\eta_{B}\left(n\right)\right)}^{2}\}$

and then

${\left({\bar{\bar{\eta }}}_{B}\left(m⊛\left(n⊛u\right)\right)\right)}^{2}$ = ${\left({\bar{\bar{\eta }}}_{B}\left(m\right)\right)}^{2}$ = ${\left({\bar{\bar{\eta }}}_{B}\left(n\right)\right)}^{2}$ = ${\left({\bar{\bar{\eta }}}_{B}\left(u\right)\right)}^{2}\leq$ max $\{{\left({\bar{\bar{\eta }}}_{B}\left(m\right)\right)}^{2}$ , ${\left({\bar{\bar{\eta }}}_{B}\left(n\right)\right)}^{2}\}$

Therefore, by (i) and (ii) above, $B^{P}$ = $(\eta_{B}$ , ${\bar{\bar{\eta }}}_{B})$ is Pythagorean fuzzy *DS*. of *B*.

Furthermore:

(a)
$a_{B}\left(m\right)$ = ${\left(\eta_{B}\left(m\right)\right)}^{2}$ + ${\left({\bar{\bar{\eta }}}_{B}\left(m\right)\right)}^{2}$ = ${\left(\eta_{B}\left(m\right)\right)}^{2}$ + $\left(1-{\left(\eta_{B}\left(m\right)\right)}^{2}\right)$ = 1
(b) s(m) = ${\left(\eta_{B}\left(m\right)\right)}^{2}-{\left({\bar{\bar{\eta }}}_{B}\left(m\right)\right)}^{2}$ = ${\left(\eta_{B}\left(m\right)\right)}^{2}-\left(1-{\left(\eta_{B}\left(m\right)\right)}^{2}\right)$ = 2 ${\left(\eta_{B}\left(m\right)\right)}^{2}-$ 1
(c)
$\pi_{B}\left(m\right)$ = $\sqrt{1-a_{P}\left(m\right)\;}$ = 0 ◻

Proposition 3.12. Let *U* be a non-void subset of *B* such that $\chi_{U}$ is characteristic function and ${\bar{\bar{\chi }}}_{U}$ is its square deviation. Then $B^{P}$ = $(\chi_{U}$ , ${\bar{\bar{\chi }}}_{U})$ is Pythagorean fuzzy *DS*. of *B* if and only if *U* is *DS*. of *B*.

Proof. Let $\chi_{U}$ : *U* → [0, 1] be characteristic function and the square deviation ${\bar{\bar{\chi }}}_{U}$ : *U* → [0, 1] be

defined as:

$$\chi_{U}\left(x\right)=\left\{ \begin{array}{l} 1\;,\;\text{if}\;x\in U,\\ 0,\;\text{if}\;x\notin U\; \end{array}\right.\;\text{and then}\;{\left({\bar{\bar{\chi }}}_{U}\right)}^{2}\left(x\right)=\left\{ \begin{array}{l} 1\;,\;\text{if}\;x\notin U,\\ 0,\;\text{if}\;x\in U\; \end{array}\right.\;$$

Suppose $B^{P}$ = $(\chi_{U}$ , ${\bar{\bar{\chi }}}_{U})\Rightarrow {\left(\chi_{U}(\left(m⊛n\right)⊛n)\right)}^{2}\geq {\left(\chi_{U}\;\left(m\right)\right)}^{2}\;$ and ${\left({\bar{\bar{\chi }}}_{U}(\left(m⊛n\right)⊛n)\right)}^{2}\leq {\left({\bar{\bar{\chi }}}_{U}\left(m\right)\right)}^{2}\;$

Let m∈ U $\Rightarrow \chi_{U}\left(m\right)$ = 1 and ${\bar{\bar{\chi }}}_{U}\left(m\right)$ = 0

Then $\chi_{U}\left(\left(m⊛n\right)⊛n\right)\geq \chi_{U}\left(m\right)$ = 1 and ${\bar{\bar{\chi }}}_{U}\left(\left(m⊛n\right)⊛n\right)\leq {\bar{\bar{\chi }}}_{U}\left(m\right)$ = 0

But $\chi_{U}\left(\left(m⊛n\right)⊛n\right)\leq$ 1 and ${\bar{\bar{\chi }}}_{U}\left(\left(m⊛n\right)⊛n\right)\geq$ 0

$\Rightarrow \chi_{U}\left(\left(m⊛n\right)⊛n\right)$ = 1 and ${\bar{\bar{\chi }}}_{U}\left(\left(m⊛n\right)⊛n\right)$ = 0

⇒(m⊛n)⊛n)∈ *U* and the other axiom can be checked similarly.

⇒ *U* is *DS*. of *B*.

Conversely, suppose *U* is *DS*. of *B*.

We claim that $B^{P}$ = $(\chi_{U}$ , ${\bar{\bar{\chi }}}_{U})$ is Pythagorean fuzzy *DS*. of *B*, which means we need to show:

(i)
${\left(\chi_{U}\left(\left(m⊛n\right)⊛n\right)\right)}^{2}\geq \chi_{U}{\left(m\right)}^{2}\;$ and ${\left(\;{\bar{\bar{\chi }}}_{U}\left(\left(m⊛n\right)⊛n\right)\right)}^{2}\leq {\left(\;{\bar{\bar{\chi }}}_{U}\left(m\right)\right)}^{2}$

  For the following we follow steps without squaring as it holds since each are members of [0, 1]
  and the result is either 1 or 0
(ii)
$\chi_{U}(m⊛\left(n⊛u\right)\geq \min\left\{\chi_{U}\left(m\right),\chi_{U}\left(n\right)\right\}\;$ and ${\bar{\bar{\chi }}}_{U}(m⊛\left(n⊛u\right)\leq \max\left\{{\bar{\bar{\chi }}}_{U}\left(m\right),\chi_{U}\left(n\right)\right\}$

(i) Now we prove this case by taking the following three cases:

Case (1) Let m∈ *U* (⇒(m⊛n)⊛n∈U by hypothesis )

$\Rightarrow \chi_{U}\left(\left(m⊛n\right)⊛n\right)$ = $\chi_{U}\left(m\right)$ = 1 $\Rightarrow \chi_{U}\left(\left(m⊛n\right)⊛n\right)\geq \chi_{U}\left(m\right)\;$ and

$\Rightarrow {\bar{\bar{\chi }}}_{U}\left(\left(m⊛n\right)⊛n\right)$ = ${\bar{\bar{\chi }}}_{U}\left(m\right)$ = 0 $\Rightarrow {\bar{\bar{\chi }}}_{U}\left(\left(m⊛n\right)⊛n\right)\leq {\bar{\bar{\chi }}}_{U}\left(m\right)$

Case (2) Let m∉ *U* and (m⊛n)⊛n∈U

$\Rightarrow \chi_{U}\left(\left(m⊛n\right)⊛n\right)$ = 1 and $\chi_{U}\left(m\right)$ = 0 $\Rightarrow \chi_{U}\left(\left(m⊛n\right)⊛n\right)\geq \chi_{U}\left(m\right)\;$ and

${\bar{\bar{\chi }}}_{U}\left(\left(m⊛n\right)⊛n\right)$ = 0 and ${\bar{\bar{\chi }}}_{U}\left(m\right)$ = 1 $\Rightarrow {\bar{\bar{\chi }}}_{U}\left(\left(m⊛n\right)⊛n\right)\leq {\bar{\bar{\chi }}}_{U}\left(m\right)$

The above steps hold for squaring (for Pythagorean) each term (and the steps hereunder also hold)

Case (3) Let m∉ *U* (m⊛n)⊛n∉U

$\Rightarrow \chi_{U}\left(\left(m⊛n\right)⊛n\right)$ = $\chi_{U}\left(m\right)$ = 0 $\Rightarrow \chi_{U}\left(\left(m⊛n\right)⊛n\right)\geq \chi_{U}\left(m\right)\;$ and

$\Rightarrow {\bar{\bar{\chi }}}_{U}\left(\left(m⊛n\right)⊛n\right)$ = ${\bar{\bar{\chi }}}_{U}\left(m\right)$ = 1 $\Rightarrow {\bar{\bar{\chi }}}_{U}\left(\left(m⊛n\right)⊛n\right)\leq {\bar{\bar{\chi }}}_{U}\left(m\right)$

(ii) By following similar steps for this as ( *i*) above, where here the cases are:

Case (1) For m , n∈ *U* (⇒m⊛(n⊛u)∈U by hypothesis )

Case (2) For (m∉ *U* or n∉ *U* ) and (m⊛n)⊛n∈U

Case (3) For (m∉ *U* or n∉ *U* ) and (m⊛n)⊛n∉U

we arrive at the result:

$\chi_{U}(m⊛\left(n⊛u\right)\geq \min\left\{\chi_{U}\left(m\right),\chi_{U}\left(n\right)\right\}\;$ and ${\bar{\bar{\chi }}}_{U}(m⊛\left(n⊛u\right)\leq \max\left\{{\bar{\bar{\chi }}}_{U}\left(m\right),\chi_{U}\left(n\right)\right\}$

Therefore, $B^{P}$ is Pythagorean fuzzy *DS*. of *B*. ◻

Theorem 3.13. The intersection, $B_{1}\cap B_{2}$ , of any two Pythagorean fuzzy *DS.s*, $B_{1}$ and $B_{2}$ , of *B* is also a Pythagorean fuzzy *DS*. of *B*.

Proof. Let $B_{1}$ = $(\eta_{B_{1}}$ , $\tau_{B_{1}})$ and $B_{2}$ =( $\eta_{B_{2}}$ , $\tau_{B_{2}})$ be any two Pythagorean fuzzy *DSs*. of *B*.

Then we need to prove that $B_{1}\cap B_{2}$ is a Pythagorean fuzzy *DS*. of *B*. Let m , n , u∈ *B*.

(i) ${\left(\eta_{B_{1}\cap B_{2}}\left(\left(m⊛n\right)⊛n\right)\right)}^{2}$ = min $\{{\left(\eta_{B_{1}}\left(\left(m⊛n\right)⊛n\right)\right)}^{2}$ , ${\left(\eta_{B_{2}}\left(\left(m⊛n\right)⊛n\right)\right)}^{2}$ }

≥ min $\{{\left(\eta_{B_{1}}\left(m\right)\right)}^{2}$ , ${\left(\eta_{B_{2}}\left(m\right)\right)}^{2}\}$ = ${\left(\eta_{B_{1}\cap B_{2}}\left(m\right)\right)}^{2}\;$ and

$\;{\left(\tau_{B_{1}\cap B_{2}}\left(\left(m⊛n\right)⊛n\right)\right)}^{2}$ = max $\{{\left(\tau_{B_{1}}\left(\left(m⊛n\right)⊛n\right)\right)}^{2}$ , ${\left(\tau_{B_{2}}\left(\left(m⊛n\right)⊛n\right)\right)}^{2}\}$

≤ max $\left\{{\left(\tau_{B_{1}}\left(m\right)\right)}^{2},{\left(\tau_{B_{2}}\left(m\right)\right)}^{2}\right\}$ = ${\left(\tau_{B_{1}\cap B_{2}}\left(m\right)\right)}^{2}$

(ii) ${\left(\eta_{B_{1}\cap B_{2}}\left(m⊛\left(n⊛u\right)\right)\right)}^{2}$ = min $\{{\left(\eta_{B_{1}}\left(m⊛\left(n⊛u\right)\right)\right)}^{2}$ , ${\left(\eta_{B_{2}}\left(m⊛\left(n⊛u\right)\right)\right)}^{2}\}$

≥ min { min $\{{\left(\eta_{B_{1}}\left(m\right)\right)}^{2}$ , ${\left(\eta_{B_{1}}\left(n\right)\right)}^{2}\}$ , min $\{{\left(\eta_{B_{2}}((m))\right)}^{2}$ , ${\left(\eta_{B_{2}}(n)\right)}^{2}\}\}$

= min { min $\{{\left(\eta_{B_{1}}\left(m\right)\right)}^{2}$ , ${\left(\eta_{B_{2}}((m))\right)}^{2}$ , min $\left\{{\left(\eta_{B_{1}}\left(n\right)\right)}^{2}\right\}$ , ${\left(\eta_{B_{2}}(n)\right)}^{2}\}\}$

= min $\{{\left(\eta_{B_{1}\cap B_{2}}\left(m\right)\right)}^{2}$ , ${\left(\eta_{B_{1}\cap B_{2}}(n)\right)}^{2}\}$ and

Similarly, we have: ${\left(\tau_{B_{1}\cap B_{2}}\left(m⊛\left(n⊛u\right)\right)\right)}^{2}\leq$ max $\{{\left(\eta_{B_{1}\cap B_{2}}(m)\right)}^{2}$ , ${\left(\eta_{B_{1}\cap B_{2}}\left(n\right)\right)}^{2}\}$ and

Hence by (i) and (ii) above, $B_{1}\cap B_{2}$ is a Pythagorean fuzzy *DS*. of *B*.

The above theorem can also be generalized to any set of Pythagorean fuzzy *DS.s* as in the following corollary. ◻

Corollary 3.14. The intersection, ${⋂}_{i\in I}B_{i}$ , of any family of Pythagorean fuzzy *DSs.*,

$\left\{B_{i}:i\in I\right\}$ , in *B* is also a Pythagorean fuzzy *DS*. of *B*, where,

$${⋂}_{i\in I}{\left(\eta_{B_{i}}\left(m\right)\right)}^{2}=\inf_{i\in I}{\left(\eta_{B_{i}}\left(m\right)\right)}^{2}\;\text{and}\;{⋂}_{i\in I}{\left(\tau_{B_{i}}\left(m\right)\right)}^{2}=\sup_{i\in I}{\left(\tau_{B_{i}}\left(m\right)\right)}^{2}.$$

Remark 3.15. The union of any two Pythagorean fuzzy DS.s of B is not necessarily Pythagorean fuzzy DS. of *B*.

Example 3.16. Let ( *B*; ⊛ , 0) be a BCL-algebra, where *B* = { *p*, *q*, *r*, 0} and let “ ⊛ ” be as defined in the Table 1 at the bottom, and define two Pythagorean fuzzy *DSs*.

$B_{1}$ = $\left({\left(\eta_{B_{1}}\right)}^{2},{\left(\tau_{B_{1}}\right)}^{2}\right)$ and $B_{2}$ = $({\left(\eta_{B_{2}}\right)}^{2},{\left(\tau_{B_{2}}\right)}^{2})\;$ as follows:

$$\;{\left(\eta_{B_{1}}\left(m\right)\right)}^{2}=\left\{ \begin{array}{l} 0.8,\;\text{if}\;m=0\\ 0.5,\;\text{if}\;m=p,q,\\ 0.1,\;\text{if}\;m=r \end{array}\right.\;\text{and}\;{\left(\tau_{B_{1}}\left(m\right)\right)}^{2}=\left\{ \begin{array}{l} 0.2,\;\text{if}\;m=0\\ 0.4,\;\text{if}\;m=p,q,\\ 0.7,\;\text{if}\;m=r \end{array}\right.$$

$$\;{\left(\eta_{B_{2}}\left(m\right)\right)}^{2}=\left\{ \begin{array}{l} 0.5,\;\text{if}\;m=0\\ 0.4,\;\text{if}\;m=p,q,\\ 0.2,\;\text{if}\;m=r \end{array}\right.\;\text{and}\;{\left(\tau_{B_{2}}\left(m\right)\right)}^{2}=\left\{ \begin{array}{l} 0.1,\;\text{if}\;m=0\\ 0.2,\;\text{if}\;m=p,q\\ 0.6,\;\text{if}\;m=r \end{array}\right.$$

It is easy to check that $B_{1}$ and $B_{2}$ are Pythagorean fuzzy DS.s of *B* but to show that their union is not necessarily a Pythagorean fuzzy DS. of *B*, we justify it as follows using the above pairs of Pythagorean fuzzy *DS.s* of *B* which are $B_{1}$ and $B_{2}$ , where ( *B*; ⊛ , 0 ) is as defined in Table at the bottom:

$$\;{\left(\eta_{B_{1}\cup B_{2}}\left(m\right)\right)}^{2}=\left\{ \begin{array}{l} 0.8,\;\text{if}\;m=0\\ 0.5,\;\text{if}\;m=p,q,\\ 0.2,\;\text{if}\;m=r \end{array}\right.\;\text{and}\;{\left(\tau_{B_{1}\cup B_{2}}\left(m\right)\right)}^{2}=\left\{ \begin{array}{l} 0.1,\;\text{if}\;m=0\\ 0.2,\;\text{if}\;m=p,q\\ 0.6,\;\text{if}\;m=r \end{array}\right.$$

Take m = r , n = p and u = r

⇒(q⊛(p⊛r)) = (q⊛p)) = r Then

(i) ${\left(\eta_{B_{1}\cup B_{2}}\left(q⊛\left(p⊛r\right)\right)\right)}^{2}$ = ${\left(\eta_{B_{1}\cup B_{2}}(r)\right)}^{2}$ = 0.2

≥ min $\left\{{\left(\eta_{B_{1}\cup B_{2}}(p)\right)}^{2},{\left(\eta_{B_{1}\cup B_{2}}(q)\right)}^{2}\right\}$

= min{0.5, 0.5 } = 0.5 is not true.

Thus, by the above justifications, the union of any two Pythagorean fuzzy DS.s of *B* is not necessarily a Pythagorean fuzzy DS. of *B*.

Theorem 3.17. Let η, be a fuzzy set such that $\eta_{B}$ is a membership function and ${\bar{\bar{\eta }}}_{B}$ is its square deviation in *B*. Suppose ${\left(\eta_{B}^{b}\right)}^{2}$ = { *x* ∈ *B*: ${\left(\eta_{B}\left(x\right)\right)}^{2}\geq {\left(\eta_{B}\left(b\right)\right)}^{2}$ ∀b∈ *B* } and then

$${\left({\bar{\bar{\eta }}}_{B}^{b}\right)}^{2}=\left\{x\in B:{\left({\bar{\bar{\eta }}}_{B}\left(x\right)\right)}^{2}\leq {\left({\bar{\bar{\eta }}}_{B}\left(b\right)\right)}^{2},\forall b\in B\right\}.$$

Then $B^{P}$ = $(\eta_{B}$ , ${\bar{\bar{\eta }}}_{B})$ is Pythagorean fuzzy *DS*. of *B* iff $B_{b}^{P}$ = $(\eta_{B}^{b}$ , ${\bar{\bar{\eta }}}_{B}^{b})$ is Pythagorean fuzzy *DS*. of *B*.

Proof. Suppose $B^{P}$ *=* $(\eta_{B}$ *,* ${\bar{\bar{\eta }}}_{B})$ is Pythagorean fuzzy *DS*. of *B*, or ∀m *,* n *,* u *,* ∈ *B*:

We need to show that $B_{b}^{P}$ *=* $(\eta_{B}^{b}$ *,* ${\bar{\bar{\eta }}}_{B}^{b})$ is Pythagorean fuzzy *DS*. of *B*.

(1) For $m\in {\left(\eta_{B}^{b}\right)}^{2}$ *;* ${\left(\eta_{B}\left(\left(m⊛n\right)⊛n\right)\right)}^{2}\geq {\left(\eta_{B}\left(m\right)\right)}^{2}\geq {\left(\eta_{B}\left(b\right)\right)}^{2}$ , and ${\left({\bar{\bar{\eta }}}_{B}\left(\left(m⊛n\right)⊛n\right)\right)}^{2}\leq {\left({\bar{\bar{\eta }}}_{B}\left(m\right)\right)}^{2}\leq {\left({\bar{\bar{\eta }}}_{B}\left(b\right)\right)}^{2}$ *;* ∀b∈ *B*
(2) *For* m *,* $n\in {\left(\eta_{B}^{b}\right)}^{2}$ *;* ${\left(\eta_{B}\left(m⊛\left(n⊛u\right)\right)\right)}^{2}\geq$ min $\{{\left(\eta_{B}\left(m\right)\right)}^{2}$ *,* ${\left(\eta_{B}(n)\right)}^{2}\}\geq {\left(\eta_{B}(b)\right)}^{2}$ and $\;{\left({\bar{\bar{\eta }}}_{B}\left(m⊛\left(n⊛u\right)\right)\right)}^{2}\leq$ max $\{{\left({\bar{\bar{\eta }}}_{B}\left(m\right)\right)}^{2}$ *,* ${\left({\bar{\bar{\eta }}}_{B}(n)\right)}^{2}$ } $\leq {\left(\eta_{B}(b)\right)}^{2}$ *;* ∀b∈ *B*

Therefore, $P^{b}$ *=* $(\eta_{B}^{b}$ *,* ${\bar{\bar{\eta }}}_{B}^{b})$ is a Pythagorean fuzzy *DS*. of *B*.

Conversely, suppose ${\left(\eta_{B}^{b}\right)}^{2}$ *=* {x∈ *B:* ${\left(\eta_{B}\left(x\right)\right)}^{2}\geq {\left(\eta_{B}\left(b\right)\right)}^{2}$ *,* ∀b∈ *B* } *and*

${\left({\bar{\eta }}_{B}^{b}\right)}^{2}$ *=* {x∈ *B:* ${\left({\bar{\eta }}_{B}\left(x\right)\right)}^{2}\leq {\left({\bar{\eta }}_{B}\left(b\right)\right)}^{2}$ *,* ∀b∈ *B* } such that $B_{B}^{P}$ *=* $(\eta_{B}^{b}$ *,* ${\bar{\eta }}_{B}^{b})$ is a Pythagorean fuzzy *DS*. of *B*.

We need to prove that $B^{P}$ *=* $(\eta_{B}$ *,* ${\bar{\bar{\eta }}}_{B})$ is Pythagorean fuzzy *DS*. of *B*.

By the hypothesis we have the following:

(1)
$m\in {\left(\eta_{B}^{b}\right)}^{2}\Rightarrow \left(\left(m⊛n\right)⊛n\right)\in {\left(\eta_{B}^{b}\right)}^{2}$ and $m\in {\left({\bar{\bar{\eta }}}_{B}^{b}\right)}^{2}\Rightarrow \left(\left(m⊛n\right)⊛n\right)\in {\left({\bar{\bar{\eta }}}_{B}^{b}\right)}^{2}$ :

$\Rightarrow {\left(\eta_{B}\left(\left(m⊛n\right)⊛n\right)\right)}^{2}\geq {\left(\eta_{B}\left(b\right)\right)}^{2}$ , ${\left({\bar{\bar{\eta }}}_{B}\left(\left(m⊛n\right)⊛n\right)\right)}^{2}\leq {\left({\bar{\bar{\eta }}}_{B}\left(b\right)\right)}^{2}$ ; ∀b∈ *B*
(2)
$m\;n\in {\left(\eta_{B}^{b}\right)}^{2}\Rightarrow m⊛\left(n⊛u\right)\in {\left(\eta_{B}^{b}\right)}^{2}$ and m , $n\in {\left({\bar{\eta }}_{B}^{b}\right)}^{2}\Rightarrow m⊛\left(n⊛u\right)\in {\left({\bar{\eta }}_{B}^{b}\right)}^{2}$

$\Rightarrow {\left(\eta_{B}^{b}(m⊛\left(n⊛u\right))\right)}^{2}\geq {\left(\eta_{B}\left(b\right)\right)}^{2}$ , and ${\left({\bar{\bar{\eta }}}_{B}^{b}(m⊛\left(n⊛u\right))\right)}^{2}\leq {\left({\bar{\bar{\eta }}}_{B}\left(b\right)\right)}^{2}$ , ∀b∈ *B*.
Thus, by (1) & (2) above, $B^{P}$ = $(\eta_{B}$ , ${\bar{\bar{\eta }}}_{B})$ is Pythagorean fuzzy *DS*. of *B*. ◻

## Conclusion

This Paper is the result of the initiations to define DS., to fuzzify it and to introduce the concept of Pythagorean fuzzy DS. of BCL-algebra following this definition and introductions, we state and prove new theorems of Pythagorean fuzzy DS. of BCL-algebra in depth which yield new fuzzified results which have not been addressed so far. The unique classifications: accuracy function, score function, degree of indeterminacy and square deviation are also characterised under the properties of the Pythagorean fuzzy DS. of BCL-algebra along with the corresponding proofs.

## Author contribution

All authors have contributed equally to the completion and success of this manuscript at each step.

## Ethics and consent

Ethical approval and consent were not required.

## Data Availability

No data are associated with this article. No extended data.
