# Supplementary material for: Pythagorean  fuzzy deductive system of BCL-algebra
Source: F1000Res. 2025 Jan 10;14:64. [Version 1] doi: 10.12688/f1000research.159263.1 (PMC11899500; doi:10.12688/f1000research.159263.1)
Supplement: Peer review history

# Reviewer report: Reviewer response for version 1

**DOI:** 10.5256/f1000research.174962.r370004

**Published:** 2025-03-10

**Authors:** Beza Derseh

**Affiliations:** 1 Department of Mathematics, Faculty of Natural and Computational Science, Debre Markos University, Debre Markos, Ethiopia,, Bahir Dar, Ethiopia

As I have thoroughly read this paper from the very beginning to the end, the manuscript introduces the novel concept of Pythagorean fuzzy deductive systems in BCL-algebras. It presents key theoretical findings that significantly enhance the understanding of such structures by extending existing algebraic frameworks with innovative approaches. The study contributes fresh insights and highlights potential applications in mathematical research, thereby advancing the field's development and expanding its practical relevance.

Is the work clearly and accurately presented and does it cite the current literature?

Yes

If applicable, is the statistical analysis and its interpretation appropriate?

Not applicable

Are all the source data underlying the results available to ensure full reproducibility?

Yes

Is the study design appropriate and is the work technically sound?

Yes

Are the conclusions drawn adequately supported by the results?

Yes

Are sufficient details of methods and analysis provided to allow replication by others?

Yes

Reviewer Expertise:

Fuzzy algebra, Intuitionistic fuzzy structures, Pythagorean fuzzy structures (like subalgebras, ideals, filters and deductive systems), Boolean algebra, Bipolar fuzzy structures

I confirm that I have read this submission and believe that I have an appropriate level of expertise to confirm that it is of an acceptable scientific standard.

# Reviewer report: Reviewer response for version 1

**DOI:** 10.5256/f1000research.174962.r369997

**Published:** 2025-03-10

**Authors:** Alachew Amaneh Mechderso

**Affiliations:** 1 University of KabriDahar, Kabri Dahar, Ethiopia

This manuscript introduces the concept of Deductive system, Pythagorean fuzzy structure , combining Pythagoran fuzzy sets with Deductive systems  in BCL- algebras. It presents several theoretical results that enhance understanding in this area. The study contributes to the field by extending existing algebraic structures with new insights and potential applications in mathematical research.

Is the work clearly and accurately presented and does it cite the current literature?

Yes

If applicable, is the statistical analysis and its interpretation appropriate?

Not applicable

Are all the source data underlying the results available to ensure full reproducibility?

Yes

Is the study design appropriate and is the work technically sound?

Yes

Are the conclusions drawn adequately supported by the results?

Yes

Are sufficient details of methods and analysis provided to allow replication by others?

Yes

Reviewer Expertise:

Reviewer Expertise: Fuzzy Sets, Bipolar Fuzzy Sets, Bipolar Soft Sets,  Fuzzy algebra

I confirm that I have read this submission and believe that I have an appropriate level of expertise to confirm that it is of an acceptable scientific standard.
